# Numerical study on flow and heat transfer characteristics of rectangular mini-channel of interpolated double S turbulators

**DOI:** 10.1371/journal.pone.0297678

**Published:** 2024-02-15

**Authors:** Yinhang Xi, Fanmao Meng

**Affiliations:** School of Mechanical and Power Engineering, Henan Polytechnic University, Jiaozuo, China; University of Science and Technology of China, CHINA

## Abstract

In this study, we propose a new type of small-channel plug-in, the double S turbulators, for passive heat transfer enhancement to improve the flow and heat transfer performance of the fluid in the channel. In the range of Reynolds number 254.51~2545.09, under constant wall temperature heating conditions, the effects of interpolated double S turbulators with different long axial radii (1mm, 1.5mm, 2mm) on the average Nusselt number, pressure drop, total thermal resistance and field synergy number in the rectangular mini-channel were studied. The simulation results show that compared with the smooth rectangular mini-channel, after interpolating double S turbulators with different long axial radii (1mm, 1.5mm, 2mm), the average Nusselt number increased by 81.74%~101.74%, 71.29%~94.06%, 67.16%~88.48%, the total thermal resistance decreased by 45.1%~50.72%, 41.72%~48.74%, 40.28%~47.2%, and the number of field synergies increased by 85.58%~111.65%, 74.1%~102.6%, 69.64%~96.12%. At present, there are few studies on the boundary condition of constant wall temperature, and this paper supplements the research on this aspect. At the same time, the heat transfer performance of the rectangular mini-channel of the interpolated double S turbulators is stronger than that of the ordinary smooth rectangular mini-channel, which not only provides a new idea for the manufacture of micro heat dissipation equipment, but also improves the heat transfer performance of micro heat dissipation equipment and improves its work efficiency. According to the simulation data, the prediction formula of average Nusselt number and pressure drop was established by nonlinear regression method, which can be used to predict the flow and heat transfer characteristics of the rectangular mini-channel of the interpolated double S turbulators.

## 1 Introduction

With the development of technology in the high-tech field, the use of miniaturization equipment is increasing. How to enhance the heat dissipation of miniaturized equipment has become an important issue for scholars to think about and discuss. In 1981, Tuckerman and Pease [1] first proposed the micro/mini-channel heat exchanger. Lim and Lee [2] have implemented counter-current heat sinks in their research on microchannel heat sinks. Hasan et al. [3] found that channel geometry has an impact on microchannel heat exchanger performance. Zheng et al. [4] proposed a novel microchannel with trapezoidal cross-section LVG. Nemati et al. [5] proposed a method to optimize the shape profile of a channel along its length in a microchannel heat sink. Wu [6] investigated the performance of a tree-type small-channel liquid-cooled radiator. Wen et al. [7] measured the thermal properties of water/ZnO nanofluids in microchannels. Liu et al. [8] studied the visualization and heat transfer performance of microchannel flow boiling. Xiao et al. [9] studied the effect of V-shaped ribs on turbulent heat transfer in mini-channel of enhanced primary flow pattern. Rajalingam A [10] investigated the effects of microstructure in microchannel heat sinks. Su [11] used numerical simulation to study the three-dimensional heat transfer problem at the entrance of a small rectangular channel at a constant wall temperature. Yao et al. [12] studied the heat transfer and service life of the new W-type micro/small channel heat exchanger. Sharma and Khan [13] investigated the effect of variable curvature of wavy microchannels on heat transfer and flow characteristics. Compared with conventional channel heat exchanger, micro/ mini-channel heat exchanger are smaller and lighter, and the heat transfer is faster and more stable.

Because the internal plug-in technology has the advantages of easy installation, low cost and easy maintenance, it has been widely studied in the conventional channel. K Sivakumar [14] research believes that the heat transfer characteristics of interpolated triangular or circular cut torsion belt round tubes are higher than those of ordinary twisted belt round tubes interpolated. Kurnia [15] believes that the new spiral tube with interpolated torsion belt is beneficial to enhance heat transfer. Bhuiya et al. [16] found that the thermal enhancement efficiency of the interpolated perforated triple twisted belt heat exchanger tube was 1.13~1.5. Mushateta et al. [17] found that the length and broadband of the interpolated tapered torsion belt have an effect on the flow and heat transfer of the heat exchanger tube. Moya-Rico et al. [18] found that interpolating twisted bands with regular spacing increased heat transfer rates. Chu et al. [19] said that the Nusselt number and frictional resistance coefficient of the interpolated obtuse V-section torsion belt were significantly improved. Feng [20] and other studies have shown that the mini-channel heat sink with interpolated torsion belt can improve its heat transfer performance and pressure drop. Chang et al. [21] proposed an innovative twisted ribbon fin array that has higher passive heat transfer enhancement and thermal performance factor for the same pressure drop loss and pumping power than the common pinfin channel. Khoshvaght-Aliabadi et al. [22] found that the thermal performance of channels inserted into novel triangular wavelet bands (DWTs) was better than that of channels inserted into homogeneous bands. Ali et al. [23] used conjugate heat transfer and computational fluid dynamics models in the range of Reynolds number 100~500 and found that the channels with interpolated tape had better thermal performance than those without tape. Smaisim et al. [24] and Song [25] studied the effects of rotating twist belts on fluid flow and heat transfer in tubes, and found that the Nussell number and pumping power increased with the increase of Reynolds number. Kosker et al. [26] found that when the Reynolds number is 5840~30900, the Nussel number increases with the increase of the curve ratio of different twist belts, and decreases with the decrease of the Reynolds number and torque ratio. Fang et al. [27] found that the heat transfer characteristics of rectangular microchannels inserted into the turbulators are stronger than those of smooth rectangular mini-channel. The nonlinear regression method was used to establish the prediction formulas of pressure drop and average Nussel number. It can be seen that the addition of internal plug-ins has a good effect on improving the heat transfer performance of micro/mini-channel.

Most of the internal plug-in technologies studied by scholars before are used for conventional channels, and there are very few internal plug-in studies for mini-channel. In this paper, double S turbulators with different long axial radii are inserted into rectangular mini-channel, their flow and heat transfer characteristics are studied, and the prediction formula of average Nusselt number and pressure drop is obtained through nonlinear regression analysis, which provides a prediction method for general conditions.

## 2 Methods and models

In this study, we use the continuum hypothesis, that is, in the simulation, it is assumed that the space through which the fluid (water) flows through a small channel can be approximated as a continuous and void-free space filled with "particles", and its macroscopic physical quantities satisfy all the physical laws that should be followed. Under this assumption, ANSYS Fluent is used to perform numerical simulations, and then part of the macroscopic physical quantities are obtained, and the data of the required parameters are calculated according to the macroscopic physical quantities. After that, the Origin graphing software is used to make a dotted line graph of the required parameters according to the data. Then, the dotted line plot of each parameter is observed, and the process and reason for its change are understood through theoretical analysis. The following studies were conducted based on this method.

The simulation method uses computational fluid dynamics (CFD) simulation, using the finite volume method to discretize the continuous, momentum, and energy conservation equations based on boundary conditions. Gambit 2.4.6 was used to generate a structured mesh of solid and fluid regions, and the mesh was verified. ANSYS Fluent was used to solve the governing equations for heat transfer and flow properties of interpolated double-S turbulators rectangular mini-channel. The SIMPLEC algorithm is used to couple the pressure and velocity equations, and the second-order style formula is used to discretize the convection term and diffusion term of the momentum equation. The convergence criterion is when the normalized residuals of all variables in the continuity, momentum, and energy equations are less than 10^−6^.

**Fig 1:**
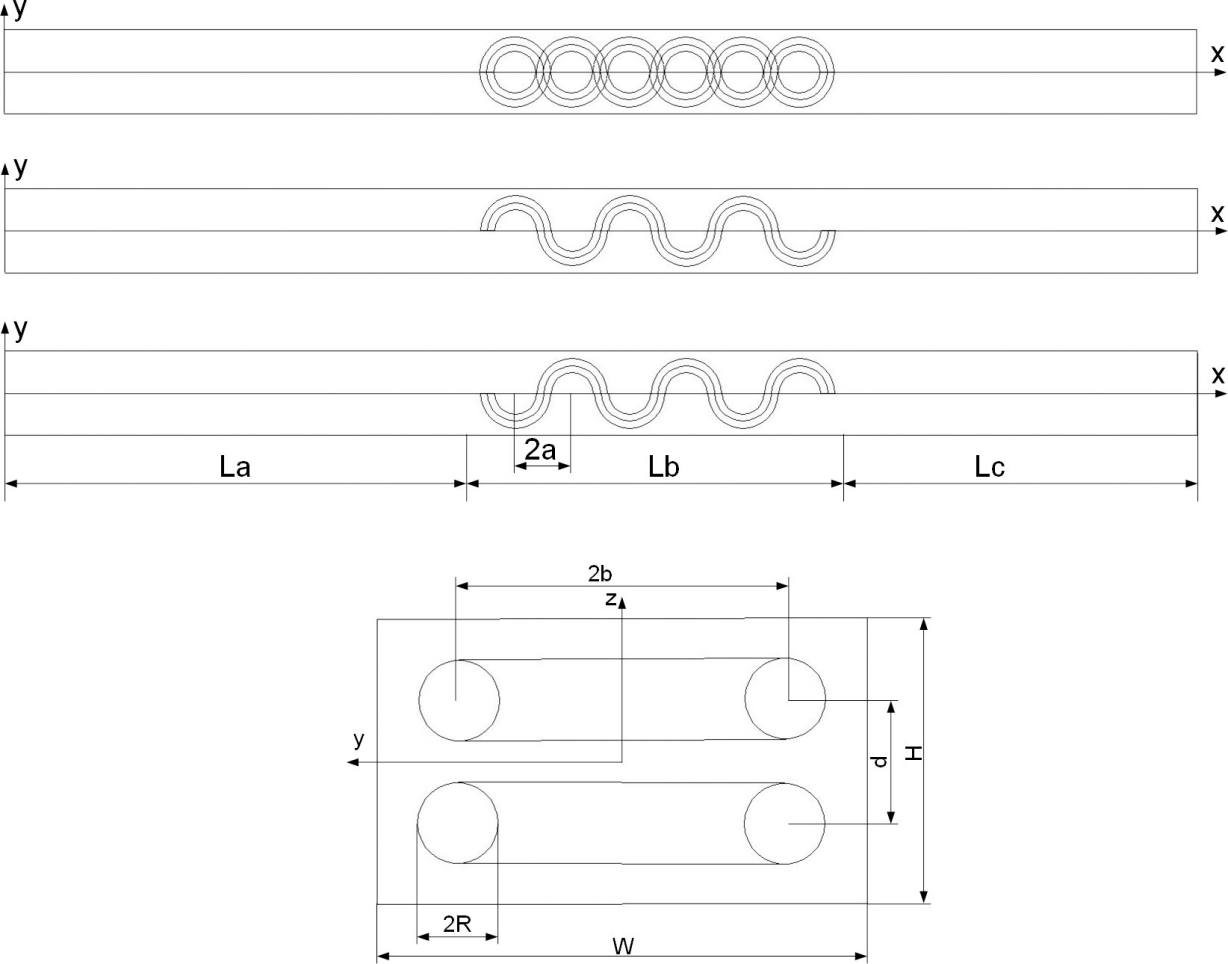
Schematic diagram of the rectangular mini-channel structure with an interpolated double S turbulators. (a) Top view, (b) Bottom view at z = 0, (c) Top view at z = 0, and (d) Side view.

A single S turbulator is formed by bending a copper wire, and its S curve can be seen as being alternately connected by the upper semicircle and lower semicircle of an ellipse (Fig 1B). Each elliptic semicircle of the S curve is a minor axis in the y direction and a long axis in the x direction. The rectangular channel interpolates two S turbulators stacked on top and bottom, the two ends of the fixture are connected and fixed with the wall surface of the small channel, but in order to facilitate the simulation, the influence of the fixture is not considered in the simulation (Fig 1D), and the overall structure is shown in Fig 1. Four specifications of rectangular channels were used in the study: (1) smooth rectangular channels (SMC); (2) Rectangular mini-channel (SMC1) with a short-axis radius of 1mm and a long-axis radius of 1mm for double S turbulators; (3) Rectangular mini-channel (SMC1.5) with a short axial radius of 1mm and a long axis radius of 1.5mm for double S turbulators; (4) Rectangular mini-channel (SMC2) with a short-axis radius of 1mm and a long-axis radius of 2mm for double S turbulators.

In Fig 1, La, Lb and Lc represent the inlet section of the mini-channel, the length of the heating section and the outlet section, a, b are the long and short axis radii of the double S turbulators, H is the height of the mini-channel, W is the width of the mini-channel, and d is the distance between the centers of the two S turbulators. Table 1 shows the structural parameters of the interpolated double S turbulators rectangular mini-channel. Table 1 Structural parameters of the double S turbulators.

**Table 1:** Structural parameters of the double S turbulators.

mini-channel type	a,mm	b,mm	La,mm	Lb,mm	Lc,mm	W,mm	H,mm	d,mm
SMC	-	-	16	13	12	3	1.75	-
SMC1	1	1	16	13	12	3	1.75	0.75
SMC1.5	1.5	1	16	13	12	3	1.75	0.75
SMC2	2	1	16	13	12	3	1.75	0.75

## 3 Thermodynamic analysis

The mini-channel and double S turbulators are made of copper, and the fluid is water. To simplify the calculation, assume that the fluid in the model is incompressible and Newtonian; The flow of the fluid is in a three-dimensional steady state; while ignoring the effects of gravity, other volumetric forces, viscous dissipation, and radiative heat transfer; The dynamic viscosity and thermal conductivity of the fluid are only related to temperature, which are physical parameters that change with temperature, and other physical parameters remain unchanged; The physical properties of solids remain constant. Based on the above assumptions, the governing equations of the fluid domain and the solid domain in Ai [28] are adopted:

Continuity equation:

∇⋅(ρU)=0 (1)

where ρ is the density of the fluid and U is the velocity vector.

Momentum equation:

∇⋅(ρUU)=ΔΡ+∇⋅(μ∇U) (2)

where P is the fluid pressure and μ is the fluid dynamic viscosity.

Energy equation:

$$\nabla \cdot (\rho CpUT_{f})=\nabla \cdot (\lambda \nabla T_{f})$$ (3)

where *Cp* is the specific heat of the fluid, T_f_ is the temperature of the fluid, and λ is the thermal conductivity of the fluid.

Equivalent diameter:

$$D_{h}=\frac{4Ac}{P}$$ (4)

Where Ac is the area of the cross section of the channel.

Inlet and outlet pressure drop:

$$\Delta P=P_{\mathrm{in}}-P_{\mathrm{out}}$$ (5)

Where P_in_ is the channel inlet pressure and P_out_ is the channel outlet pressure.

Average temperature difference between heating surface and fluid:

$$\Delta T=T_{w}-\frac{T_{in}+T_{out}}{2}$$ (6)

Where T_w_ is the channel wall temperature, T_in_ is the channel inlet temperature, and T_out_ is the channel outlet temperature.

Reynolds number:

Re=ρuinDhμ (7)

Average heat transfer coefficient:

$$h_{\mathrm{ave}}=\frac{Q}{A\Delta T}$$ (8)

Where A is the area of the heated surface and Q is the heat flux.

Average Nusselt number:

$${Nu}_{\mathrm{ave}}=\frac{h_{ave}D_{h}}{\lambda }$$ (9)

Local heat transfer coefficient:

$$h_{x}=\frac{Q}{A(T_{w,\;x}-T_{f,\;x})}$$ (10)

Local Nusselt number:

$${Nu}_{x}=\frac{h_{x}D_{h}}{\lambda }$$ (11)

Total thermal resistance:

$$R_{T}=\frac{\Delta T}{Q}$$ (12)

Co-efficient of fluid velocity vector and pressure gradient:

U∇P=|U‖∇P|cosα (13)

Where α is the synergy angle between fluid velocity vector and pressure gradient.

Synergistic relationship between fluid velocity vector and temperature gradient:

U∇T=|U‖∇T|cosβ (14)

Where β is the synergy angle between fluid velocity vector and temperature gradient.

Field co-efficient:

$$Fc=\int \left(U\cdot \nabla T\right)dy=\frac{{Nu}_{ave}}{RePr}$$ (15)

Prandtl number:

$$Pr=\frac{Cp\mu }{\lambda }$$ (16)

Dimensionless quantity:

$$x^{*}=\frac{x}{D_{h}RePr}$$ (17)

where x is the length from the channel inlet at an axial position along the flow direction in the channel.

The model in this study generated a structured mesh of solid and fluid regions from gambit 2.4.6 and solved using ANSYS Fluent software. The boundary conditions of the model are set as follows: the fluid inlet temperature T_in_ = 300K, the fluid inlet velocity is u_in_ = 0.1~1m/s, the range corresponding to the Reynolds number is 254.51~2545.09; the outlet condition of the mini-channel is free outflow; The heating temperature of the rectangular mini-channel heating section T_w_ = 330K, and the heating section wall is at a constant temperature, and the other walls are regarded as insulation, and all walls have no slippage. Each model is set to 10 working conditions with a different rectangular mini-channel inlet speed. The inlet speed of rectangular mini-channel corresponding to 10 working conditions is at intervals of 0.1m/s, from 0.1m/s to 1m/s.

**Fig 2:**
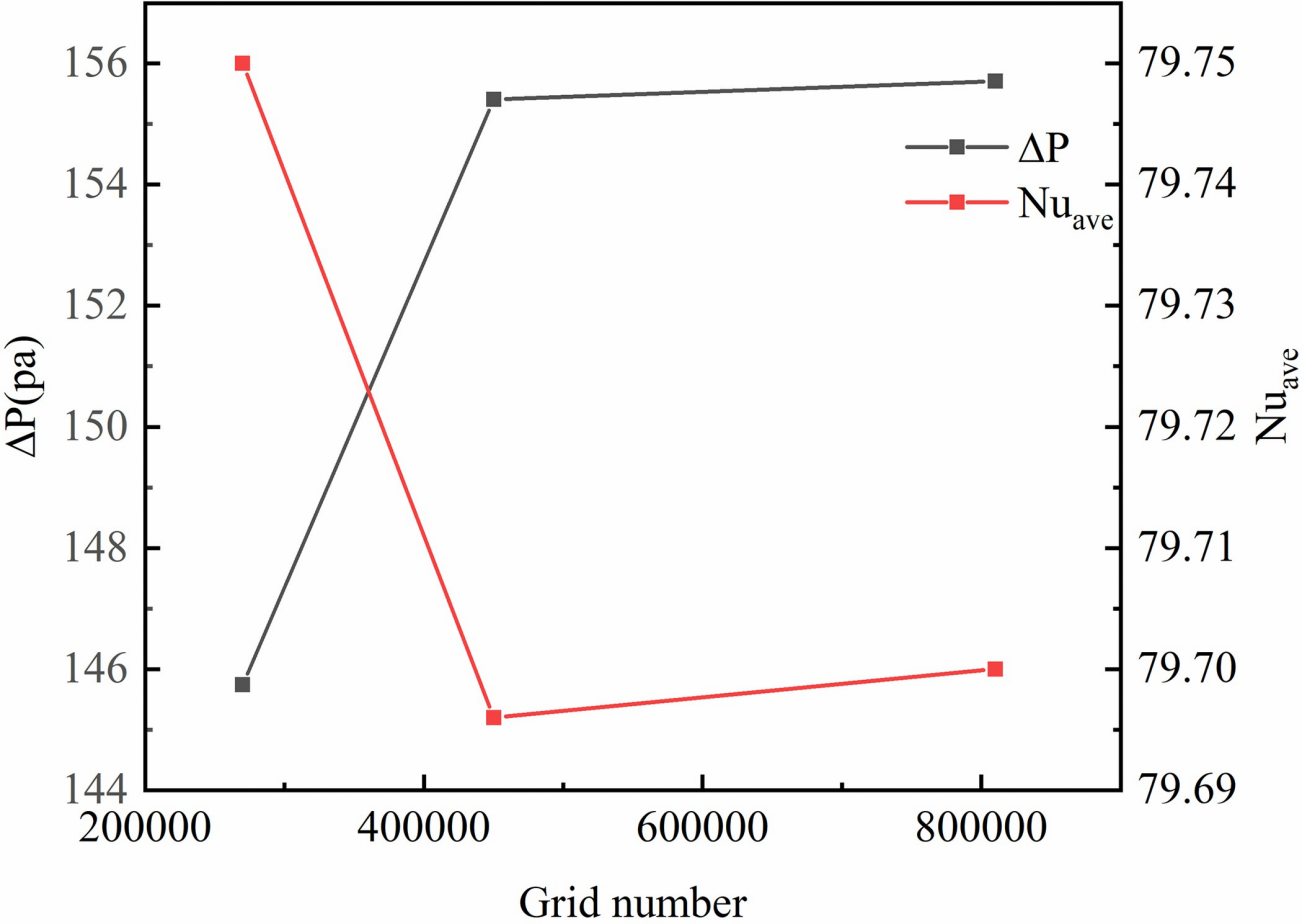
Grid validation.

To avoid the number of meshes affecting the simulated process and the results, the independence of the meshes needs to be verified. Fig 2 shows the change in pressure drop and average Nusselt number for different mesh numbers. Fig 2 shows the pressure change and average Nusselt number along the path under different mesh numbers, and it can be seen from the Fig 2 that the difference in pressure and average Nusselt number change between different mesh numbers gradually decreases as the number of grids increases. When the number of grids increases from 270,000 to 540,000, the pressure change error is about 4.6% and the change in the average Nusselt number was 0.06%. when the number of grids increases from 540,000 to 810,000, the pressure change error is less than 0.1%, the average Nusselt number changed by less than 0.01%. Considering the calculation time and resources, a grid of 540,000 was selected to divide the model.

**Fig 3:**
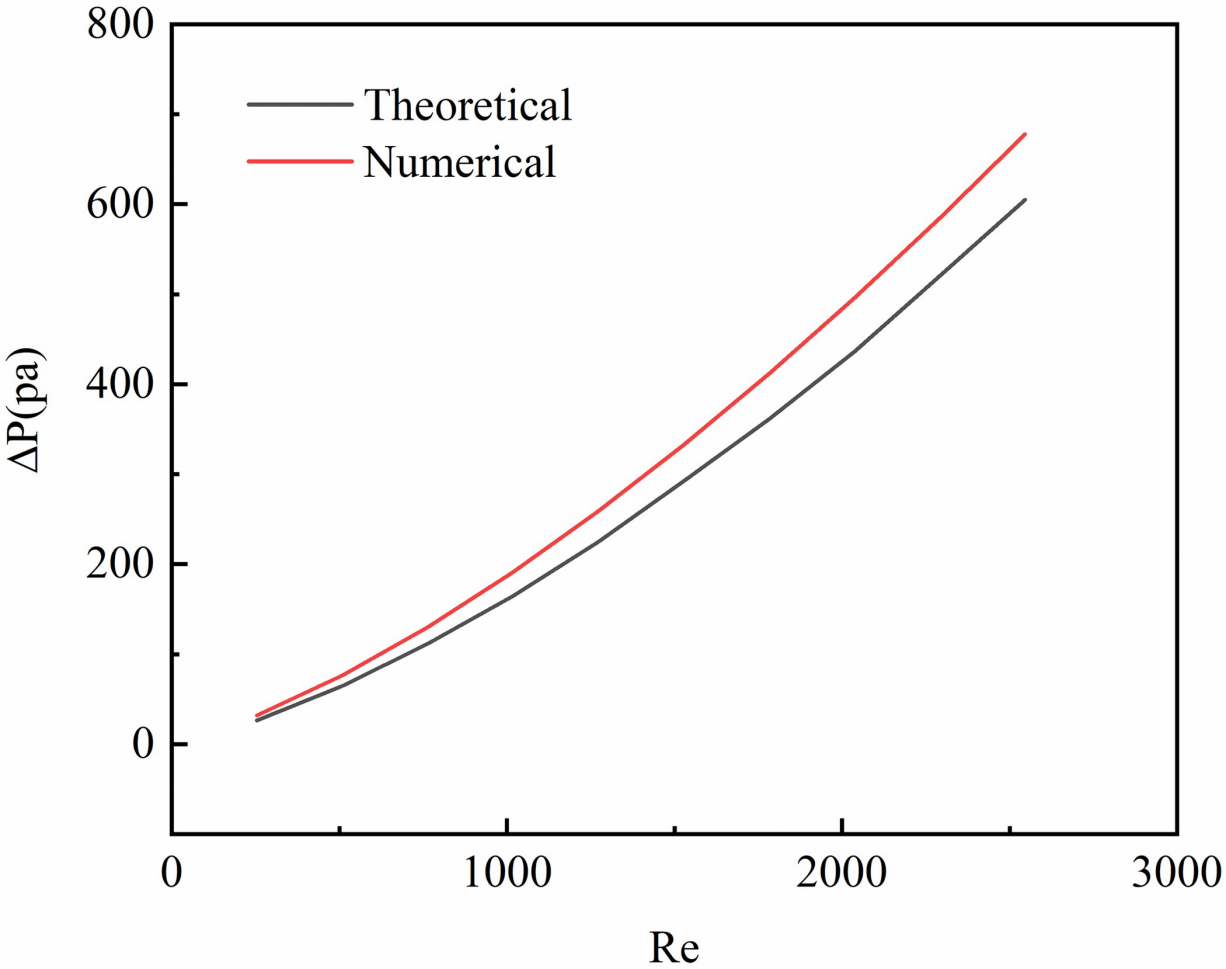
Theoretical verification.

In order to verify the reliability of the simulation, the relevant data obtained from the simulation need to be analyzed and verified. The theoretical validation method described in Ai [28] was used, that is, using the calculation results of the theoretical pressure drop calculation formula proposed by Kandlikar [29], the theoretical pressure drop calculation formula is shown in Eq 24. Comparing the results obtained by formula with the simulation results as shown in Fig 3. It can be seen from Fig 3 that the numerical calculation of the pressure drop is mostly higher than the calculation result of the pressure drop formula proposed by Kandlikar [29], and the average percentage deviation is 13.2%. This proves the reliability of the numerical simulation results.

## 4 Results analysis and discussion

**Fig 4:**
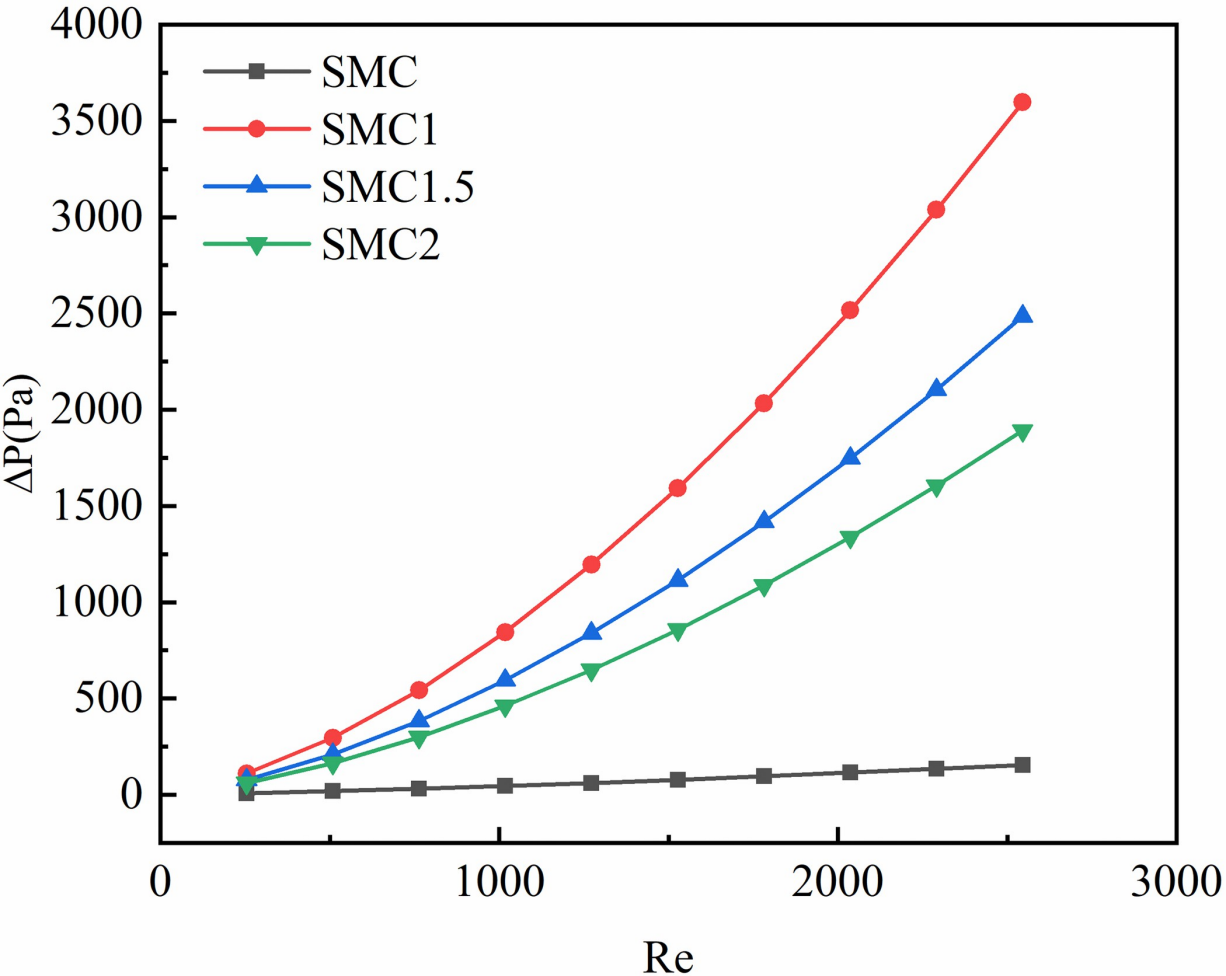
The change of pressure drop of four rectangular channels with Re.

The pressure drop is caused by the internal friction and the collision between the fluid particles in the turbulence. It is manifested by the pressure difference generated before and after the fluid flow, in (pa). Fig 4 shows the four rectangular mini-channel inlet and outlet pressure drops as a function of Reynolds number. It can be seen from Fig 4 that the inlet and outlet pressure drops of smooth rectangular mini-channel (SMC) and rectangular mini-channel (SMC1, SMC1.5, SMC2) interpolating three different long axis radii (1mm, 1.5mm, 2mm) of double S turbulators (SMC1, SMC1.5, SMC2) gradually increase with the increase of Re. Compared with the change trend of the inlet and outlet pressure drop of the smooth rectangular mini-channel, the change trend of the inlet and outlet pressure drop of the rectangular mini-channel interpolated with double S turbulators was more severe. This is because the addition of the double S turbulators significantly increases its contact area with the fluid, and because the double S turbulators itself has a large shape resistance. The addition of the double-S turbulators causes the fluid to flow radially in the channel and a flow vortex nearby, which makes the contact between the fluid and the wall of the small rectangular channel larger, so that a huge pressure drop loss is generated. In a small rectangular channel interpolated with double S turbulators, the smaller the radius of the long axis, the greater the amplitude of the inlet and outlet pressure drop with Re. Comparing the inlet and outlet voltage drop changes of the four rectangular mini-channel, it can be seen that SMC1>SMC1.5>SMC2>SMC.

**Fig 5:**
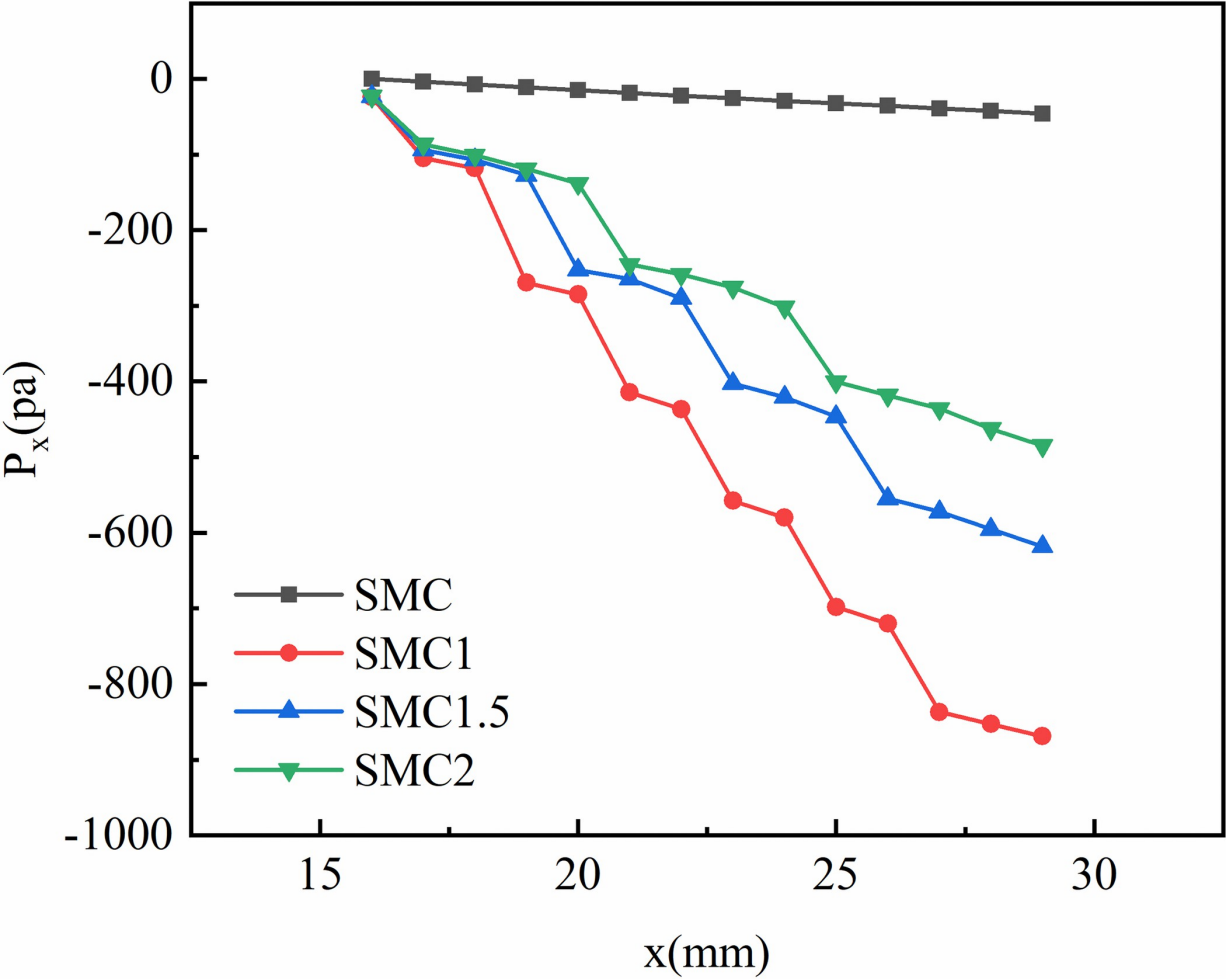
Local pressure changes in four rectangular small rectangular channels at Re = 1018.04, x = 16~29.

Fig 5 shows the change of the local pressure in the four rectangular mini-channels at Re = 1018.04, x = 16~29mm. Fig 5 shows that the local pressure of the four rectangular mini-channel decreases gradually along the direction of the flow. The SMC mini-channel pressure drops very slowly, In contrast, the pressure drop of the rectangular small channel with the interpolated double S turbulators is larger. This is because the addition of the double S turbulators significantly increases the pressure throughout the channel. At the same time, it is found that the pressure of the rectangular small channel inlet section with three interpolated double S turbulatorss with different long-axis radii is almost not reduced. At the same time, it is found that the pressure of the rectangular small channel inlet section with three interpolated double S turbulatorss with different long-axis radii is almost not reduced. Among the rectangular mini-channel with an interpolated double S turbulators, the SMC1 mini-channel has the largest pressure change, followed by SMC1.5, with a relatively small change being SMC2. This is because when the fluid reaches the same position in the rectangular small channel, the long axis radius of the interpolating double S turbulators is different, which causes the local pressure change.

**Fig 6:**
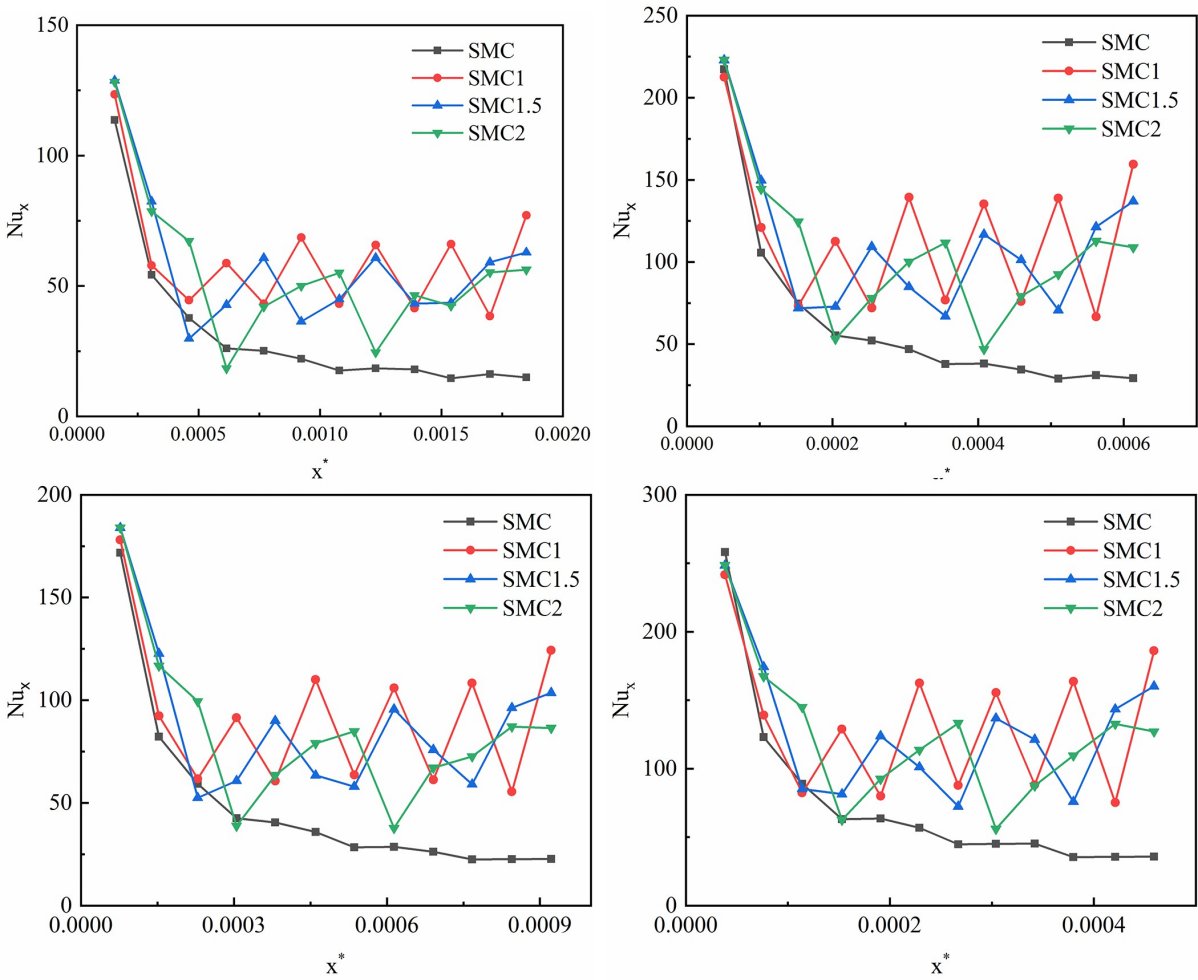
Changes of local Nusselt number (a) Re = 509.02, x* = 0~0.00185; (b) Re = 1018.04, x* = 0~0.00092; (c)Re = 1527.06, x* = 0~0.00061;(d)Re = 2036.07, x* = 0~0.0005.

Fig 6 shows the local Nusselt number of the smooth rectangular mini-channel decreases in the flow direction, and there is a rapid decrease process at the entrance, and then the decrease decreases rapidly, showing a trend of slow decrease and stabilization not far after the entrance. The local Nusselt number of the rectangular mini-channel of the interpolated double S turbulators also has a rapid decrease process at the entrance, and there is a periodic change of up and down fluctuations not far after the entrance, which is caused by the disturbance of the double S turbulators, and its overall change trend shows a stable trend. It is also found that the local Nusselt number of SMC1 fluctuates much more than SMC1.5 and SMC2, which indicates that the shorter the long axis radius of the interpolated double S turbulators has a greater effect on the local Nusselt number.

**Fig 7:**
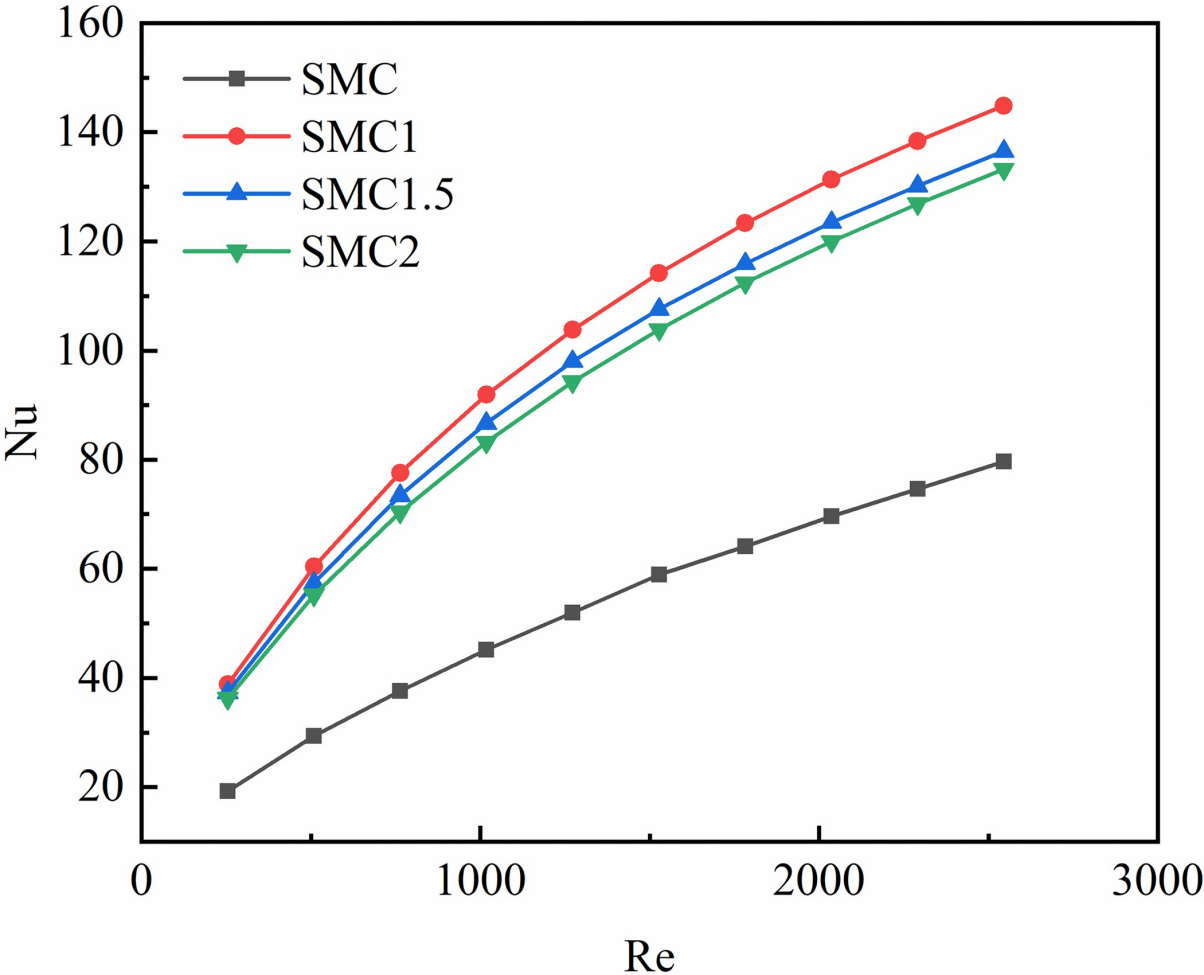
The average Nusselt number of the four rectangular mini-channels varies with Re.

The average Nusselt number is expressed as a criterion for the average degree of convective heat transfer in a small channel, and also represents the average ratio of the thermal conductivity resistance of the bottom layer of the fluid laminar flow to the average ratio of the convective heat transfer resistance, which is a dimensionless number. Fig 7 shows the average Nusselt number of the four rectangular channels increases with the increase of the Re. The average Nusselt number of smooth rectangular mini-channel is relatively small, and the increase with Re is relatively small. Compared with the smooth rectangular mini-channel, the average Nusselt number of the interpolated double S turbulators rectangular mini-channel is relatively large, and the average Nusselt number of SMC1, SMC1.5 and SMC2 increases by 81.74%~101.74%, 71.29%~94.06%, 67.16%~88.48%, respectively. At the same time, under the same Re, the size of three rectangular channels interpolated with different long axis radii is: SMC1> SMC1.5> SMC2. In summary, the addition of double S turbulators significantly increases the average Nusselt number, and the shorter the radius of the long axis, the greater the amplitude, which is more conducive to heat transfer.

**Fig 8:**
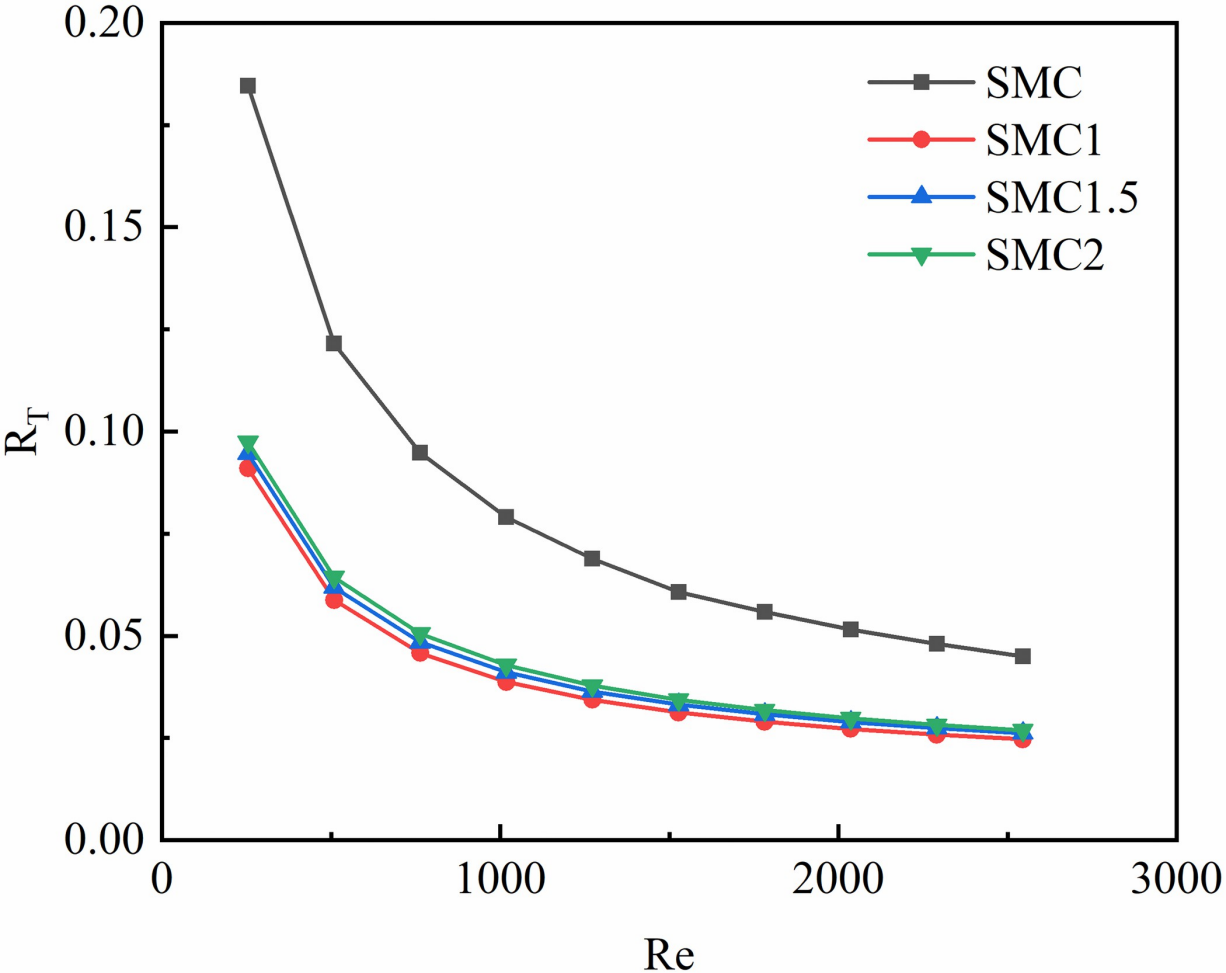
Total thermal resistance changes with the Re.

Thermal resistance is the ratio of the temperature difference between the two ends of the object and the power of the heat source when heat is transferred over the object, in units of (k/w) or (c/w). Fig 8 shows that the total thermal resistance of the four rectangular mini-channels decreases with the increase of the Re. However, the total thermal resistance of the rectangular channel of the interpolated double S turbulators is less than the total thermal resistance of the smooth rectangular channel. Compared with SMC, the total thermal resistance of SMC1, SMC1.5 and SMC2 decreased by 45.1%~50.72%, 41.72%~48.74%, 40.28%~47.2%, respectively, and the shorter the radius of the long axis, the greater the decrease. At the same time, we can find that the total thermal resistance of the three mini-channel with different long axis radii is nearly equal. This indicates that the radius length of the double S turbulators has little effect on the total thermal resistance of the rectangular small channel of the double S turbulators with different long-axis radius.

**Fig 9:**
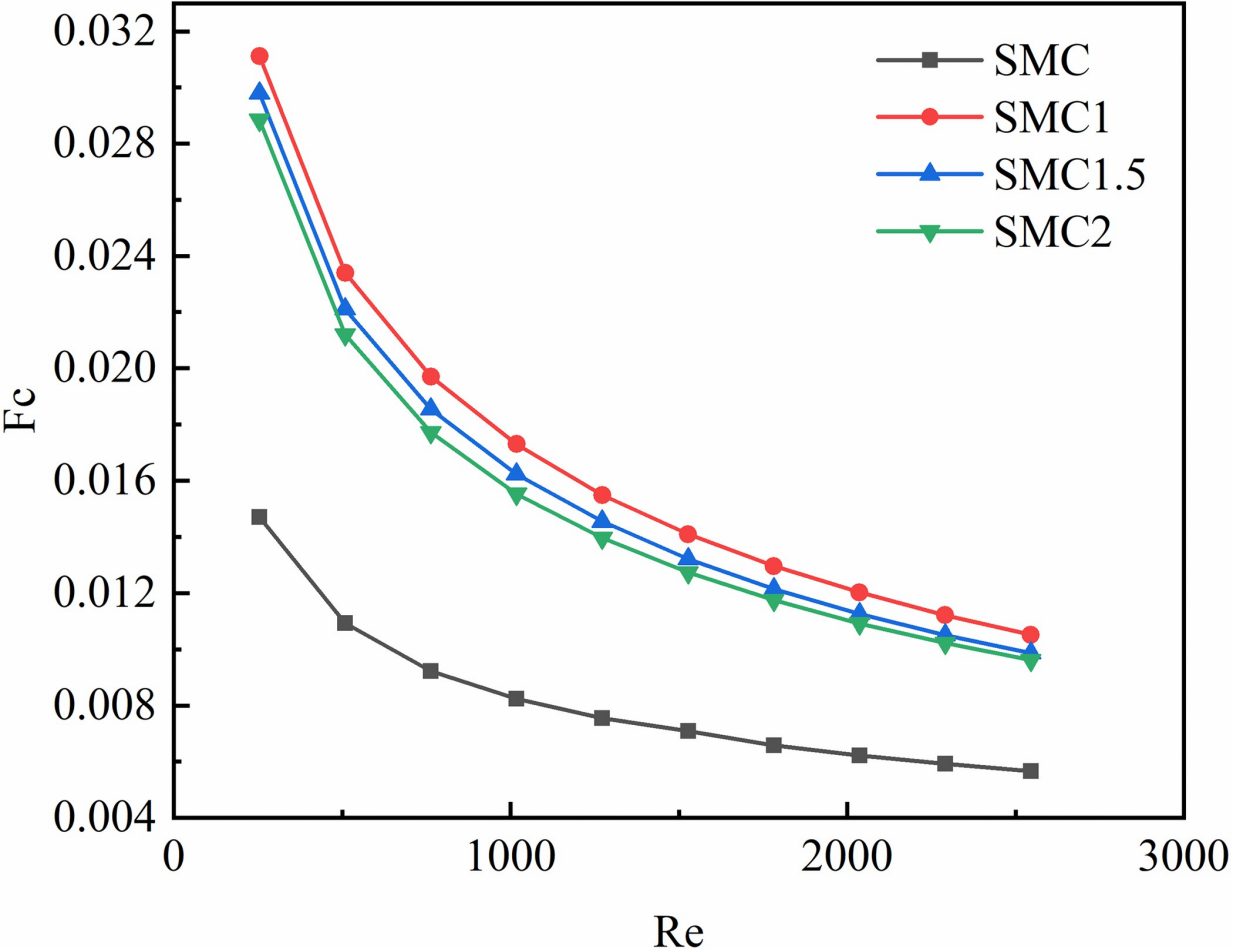
The synergy number of four rectangular mini-channels fields with Re.

Fig 9 shows the change of the cooperative number with Re. It shows the field synergy number of the four rectangular mini-channels decreases with the increase of the Re, with values of much less than 1. When the Re is small, the field synergy number decreases faster, and with the increase of the Re, the field synergy number decreases slowly. The reason is that when the physical properties of the fluid do not change much, the increase rate of the Nusselt number is much smaller than that of the Re, so the field synergy number decreases with the increase of the Re. The field synergy number of the rectangular channel of the interpolated double S turbulators is generally greater than that of the smooth rectangular mini-channel, which is due to the disturbance of the fluid by the interpolated double S turbulators, which improves the synergy between velocity and temperature gradient, reduces the angle between the two, and enhances heat transfer. Compared with SMC, the field synergy numbers of SMC1, SMC1.5 and SMC2 increased by 85.58%~111.65%, 74.1%~102.6%, 69.64%~96.12%, respectively.

Based on the above analysis, it is found that the interpolated double S turbulators makes the Nusselt number and pressure drop of the rectangular channel higher than that of the smooth rectangular channel, so the influence of these factors on the heat transfer performance needs to be comprehensively considered. In this paper, the comprehensive performance heat transfer factor (PEC) is used to evaluate the comprehensive heat transfer performance of rectangular mini-channel, which is defined as follows:

Frictional resistance coefficient:

$$f=\frac{\Delta P}{2\rho u^{2}}$$ (18)

Integrated performance heat transfer factor:

$$PEC=\frac{{Nu}_{ave}/{Nu}_{0,ave}}{{\left(f/f_{0}\right)}^{1/3}}$$ (19)

where Nu_ave_ and f are the average Nusselt number and frictional drag coefficient of double S turbulators rectangular mini-channels with different long axial radii, and Nu_0_,_ave_ and f_0_ are the average Nusselt number and frictional drag coefficient of the smooth channel under the same conditions.

**Fig 10:**
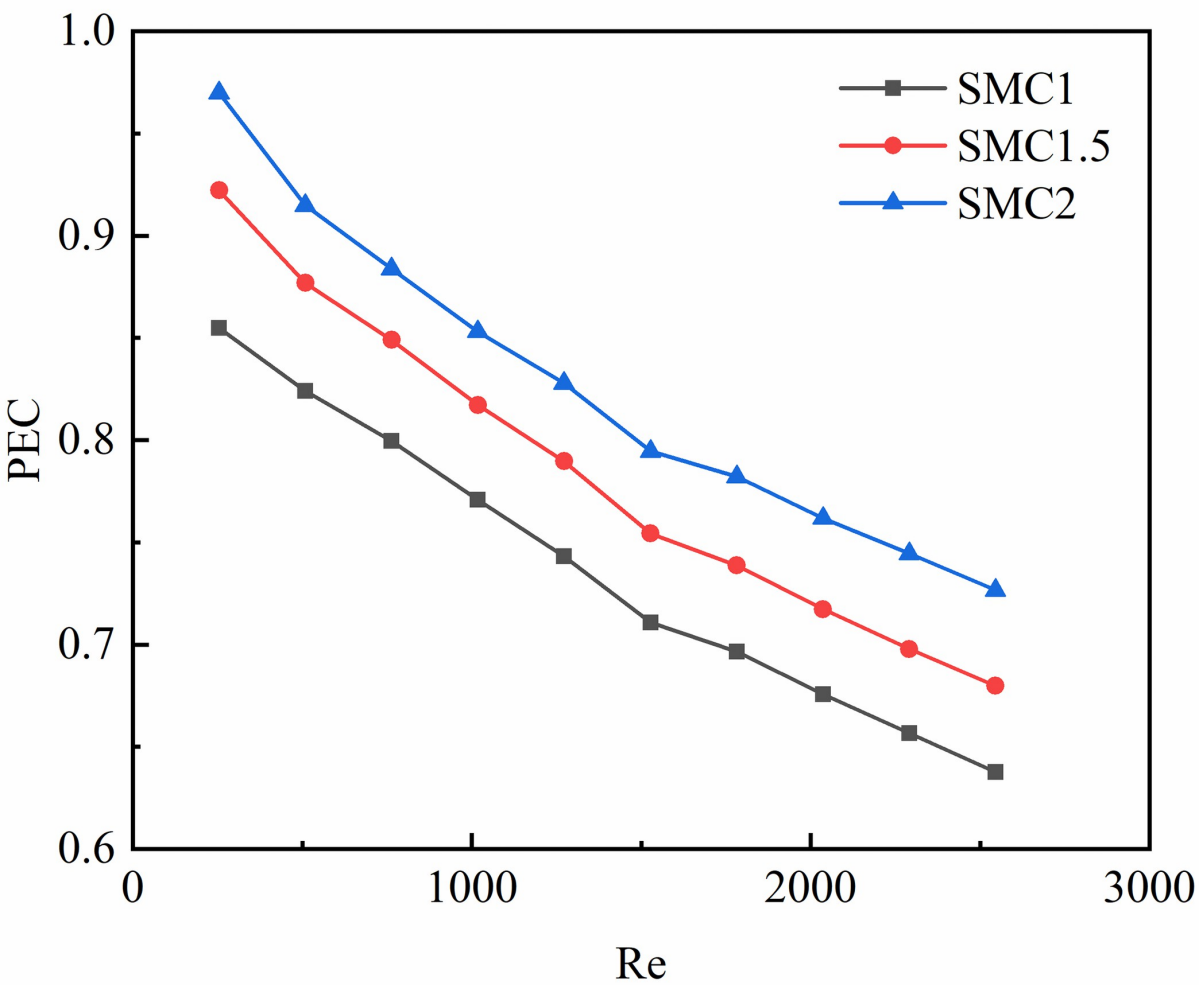
The comprehensive performance factors of three kinds of the interpolated double S turbulators rectangular mini-channels with different long axis radii vary with Re.

Fig 10 shows the PEC of the rectangular channels interpolated by double S turbulators with different long axial radii decreases with the increase of Re. PEC, SMC2> SMC1.5>SMC1 when the Re is the same. Although the average Nusselt number of SMC1 is relatively largest, the growth rate of its voltage drop is relatively large to a greater extent than the growth rate of the Nusselt average number, resulting in the largest friction drag coefficient of SMC1, so SMC1 is the smallest PEC among the three interpolated double S turbulators rectangular channels with different long axial radii. Therefore, the smaller the Re, the better the integrated heat transfer factor of the rectangular small channel.

Based on the prediction formula established in Ai [28], According to the double S turbulators parameters (a, b), Re Re and Prandtl number Pr, the prediction formula for flow and heat transfer was established by nonlinear regression method. The prediction formula for the average Nusselt number of the interpolated double S turbulators rectangular mini-channel is expressed as:

$${Nu}_{\mathrm{ave}}={1.35Re}^{0.53}{Pr}^{1/3}{\left(\frac{a}{b}\right)}^{-0.14}$$ (20)

The correlation coefficient R = 0.9985 for Nu_ave_’s prediction formula.

The pressure drop prediction equation is given in the form of the apparent friction coefficient f_app_:

ΔP=2fappReμuinLcDh2 (21)

Through the simulation results, the prediction formula of the product of the apparent friction coefficient and the Re is established, and the prediction formula of the interpolated double S turbulators rectangular mini-channel can be expressed as:

$$f_{app}Re=mx^{+n}$$ (22)

where m,n are dimensionless coefficients, as shown in Table 2:

**Table 2:** Table of dimensionless coefficients.

mini-channel	m	n
SMC1	37.65	-0.51
SMC1.5	28.66	-0.49
SMC2	24.3	-0.48

The correlation coefficient R for the prediction formula of SMC1, SMC1.5 and SMC2 is 0.9982,0.9985 and 0.9987, respectively.

**Fig 11:**
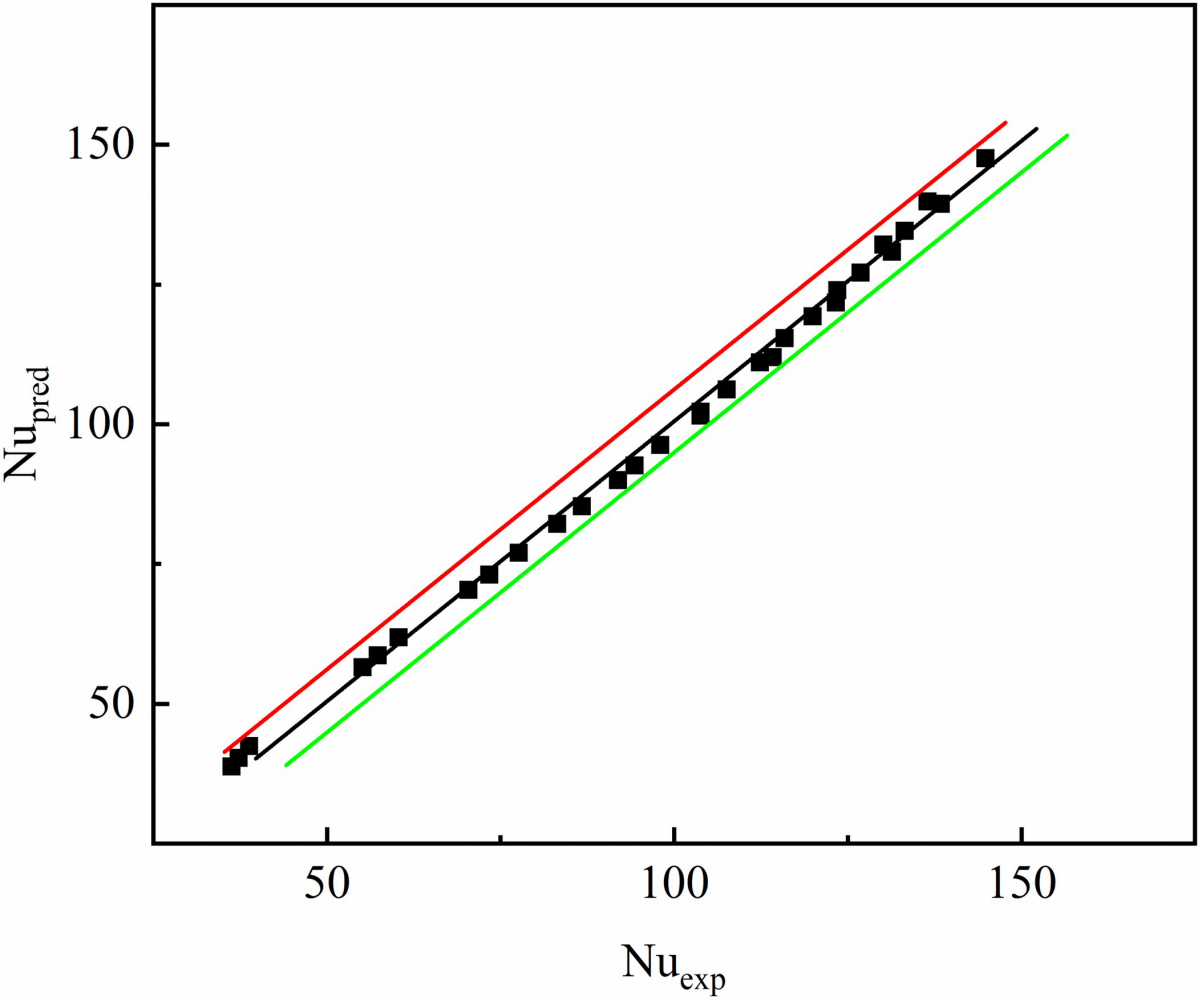
The predicted and simulated values compare.

**Fig 12:**
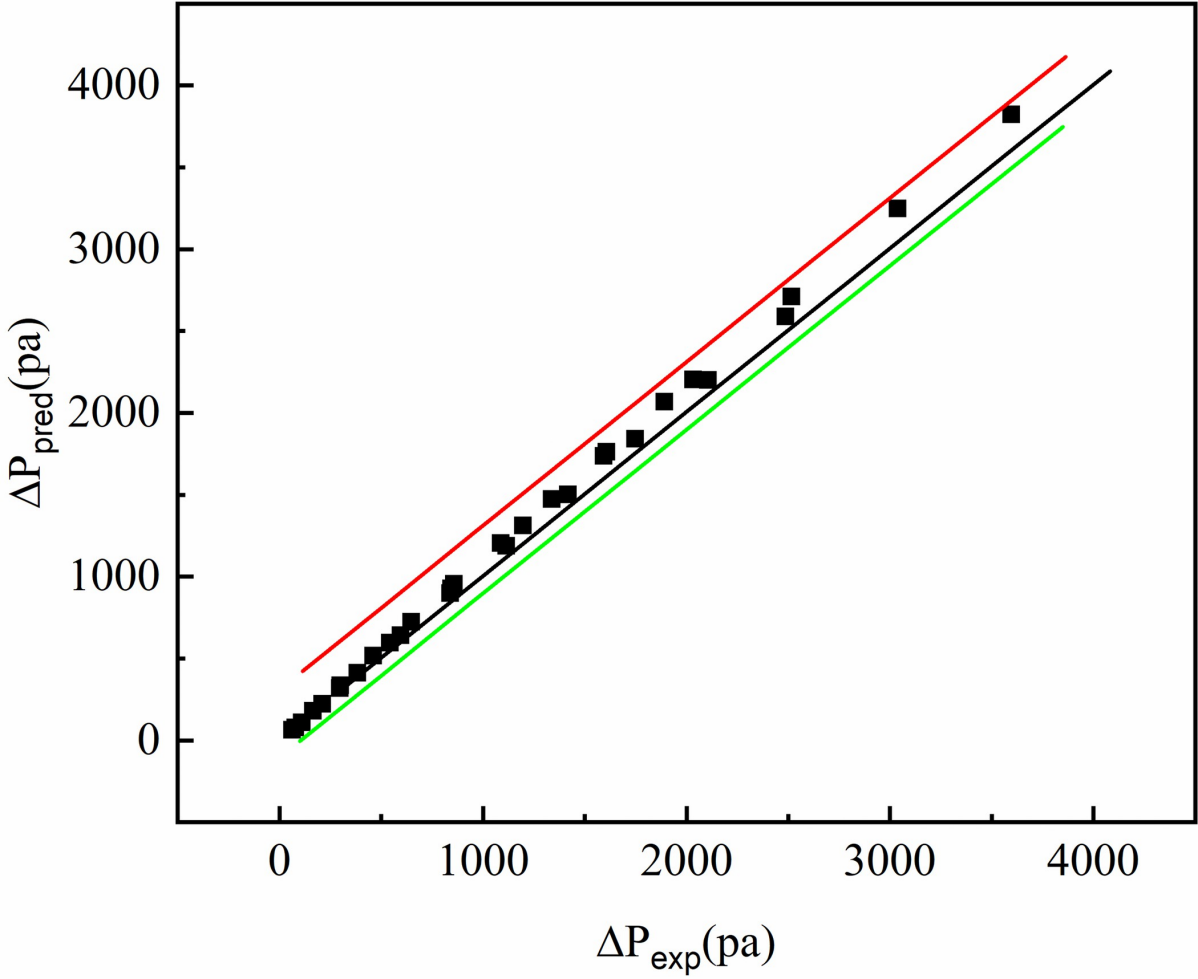
Comparison of predicted and simulated values.

Figs 11 and 12 compare the predicted values of the average Nusselt number and voltage drop of the interpolated double S turbulators rectangular channel with the analog value, respectively. As can be seen from Fig 11, the Nu_ave_ prediction values of SMC1, SMC1.5 and SMC2 do not deviate much from the analog values, and the error fluctuates between -8.59%~2.39%, and most of them are within ±2%. As can be seen from Fig 12, the deviation of the ΔP prediction value of SMC1, SMC1.5 and SMC2 from the analog value is the minimum of 0.17% and the maximum of 10.7%. It can be seen that the error of the prediction formula is acceptable.

## 5 Application prospects

In the field of heat dissipation of miniaturized equipment, mini-channel have good flow and heat transfer characteristics, so more and more experts and scholars have studied them. The internal plug-in can strengthen heat transfer and has the advantages of easy installation and low cost. In view of this, this paper studies the flow and heat transfer characteristics of the rectangular and smooth rectangular mini-channel of the internal plugin——double S turbulators, Through the simulation and comparison of the relevant parameters, it is found that the heat transfer performance of the rectangular small channel with the interpolated double S turbulators is stronger than that of the smooth rectangular small channel, which indicates that this study is meaningful. Therefore, this paper not only provides a new type of internal plug-in-in structure for the industrial production of internal plug-in (e. g., microelectromechanical systems, microsatellites, high-voltage electrical appliances). At the same time, because the heat transfer performance of the interpolated double S turbulators rectangular channel is stronger than that of the ordinary smooth rectangular channel, the interpolated double S turbulators rectangular channel can replace the ordinary smooth rectangular channel, so as to improve the heat transfer performance of micro heat dissipation equipment, improve its working efficiency and prolong its service life.

## 6 Conclusion

In this paper, the flow and heat transfer characteristics of smooth rectangular mini-channel and rectangular mini-channel interpolated with double S turbulators with different long axial radii (1mm, 1.5mm, 2mm) in the range of Re 254.51~2545.09 under constant wall temperature heating and free flow conditions are studied by numerical simulation method. The main conclusions are as follows:

1. The average Nusselt number and voltage drop of the four small rectangular channels increase with the increase of Re, while the total thermal resistance and field synergy number decrease with the increase of Re.
2. Compared with the smooth rectangular mini-channel, after interpolating double S turbulators with different long axis radii (1mm, 1.5mm, 2mm), the average Nusselt number increased by 81.74%~101.74%, 71.29%~94.06%, 67.16%~88.48%, the total thermal resistance decreased by 45.1%~50.72%, 41.72%~48.74%, 40.28%~47.2%, and the field synergy increased by 85.58%~111.65%, 74.1%~102.6%, 69.64%~96.12%, respectively.
3. When the Re is the same, the comprehensive performance heat transfer factor is SMC2>SMC1.5>SMC, and the smaller the Re, the better the comprehensive performance heat transfer factor of the rectangular small channel with the interpolated double S spoiler.
4. Based on the simulated data, the prediction formula of the average Nusselt number and pressure drop was established by nonlinear regression method, and the error was within the acceptable range.
5. In the field of heat dissipation of miniaturized equipment, the rectangular small channel of interpolated double S spoiler can be used to replace the ordinary smooth rectangular small channel, so as to improve the heat transfer performance and work efficiency of the equipment and prolong its service life.

## Supporting information

S1 Table Important data in the simulations. (DOCX)

S2 Table Local data supplement. (DOCX)

## Abbreviations

a: long axis radius of S spoiler, [mm]
b: short axis radius of S spoiler, [mm]
La: length of small channel inlet section,[mm]
Lb: length of small channel heating section,[mm]
Lc: length of small channel exit section,[mm]
W: width of small channel section, [mm]
H: height of small channel section,[mm]
d: distance between the centers of the two S spoilers,[mm]
R: radius of S spoiler,[mm]
Q: heat flow rate,[W]
u_in_: channel inlet velocity,[m/s]
C_p_: density of fluid,[kJkg^-1^k^-1^]
D_h_: equivalent diameter,[mm]
ΔP: differential pressure of channel inlet and outlet,[pa]
P_in_: channel inlet pressure,[pa]
P_out_: channel outlet pressure,[pa]
P: pressure,[pa]
ΔT: differential temperature of channel inlet and outlet,[K]
T_in_: channel inlet temperature,[K]
T_out_: channel inlet temperature,[K]
T_f_: fluid temperature,[K]
T_w_: channel wall temperature,[K]
A: heated surface area,[m^2^]
A_c_: channel cross-sectional area,[m^2^]
Nu_ave_: average Nussle number
Nu_x_: local Nussle number
H_ave_: average convective heat transfer coefficient,[Wm^-2^k^-1^]
H_x_: local convective heat transfer coefficient,[Wm^-2^k^-1^]
Re: reynolds number
R_T_: total thermal resistance,[m^2^k·kw^-1^]
Pr: prandtl number
Fc: field coefficient
PEC: integrated Performance Heat Transfer Factor
M: dimensionless coefficient
N: dimensionless coefficient
X*: dimensionless distance
Greek symbol μ: viscosity coefficient of the fluid,[Pa·s]
ρ: density of the fluid,[kgm^-3^]
λ: thermal conductivity of the fluid,[Wm^-1^K ^-1^]
α: angle between the velocity vector and the temperature gradient,[°]
β: angle between fluid velocity vector and pressure gradient,[°]
Subscripts ave: average value
f: fluid
in: channel inlet
out: channel outlet
w: channel wall
x: local
