# Supplementary material for: Numerical study on flow and heat transfer characteristics of rectangular mini-channel of interpolated double S turbulators
Source: PLoS One. 2024 Feb 15;19(2):e0297678. doi: 10.1371/journal.pone.0297678 (PMC10868837; doi:10.1371/journal.pone.0297678)
Supplement: S1 Table — (DOCX) [file pone.0297678.s001.docx]

S1 Table. Important data in the simulations

| mini-channel | **Re** | **Nu_ave_** | **∆P** | **R_T_** | **F_C_** | **PEC** |
| --- | --- | --- | --- | --- | --- | --- |
| **SMC** | 254.51 | 19.24 | 8.42 | 0.185 | 0.0147 | - |
|  | 509.02 | 29.34 | 19.01 | 0.122 | 0.0109 | - |
|  | 763.53 | 37.63 | 31.66 | 0.095 | 0.0092 | - |
|  | 1018.04 | 45.19 | 45.96 | 0.079 | 0.0082 | - |
|  | 1272.55 | 51.98 | 61.66 | 0.069 | 0.0075 | - |
|  | 1527.06 | 58.92 | 78.57 | 0.061 | 0.0071 | - |
|  | 1781.57 | 64.09 | 96.51 | 0.056 | 0.0066 | - |
|  | 2036.07 | 69.58 | 115.38 | 0.052 | 0.0062 | - |
|  | 2290.58 | 74.67 | 135.04 | 0.048 | 0.0059 | - |
|  | 2545.09 | 79.70 | 155.41 | 0.045 | 0.0057 | - |
| **SMC1** | 254.51 | 38.82 | 110.57 | 0.091 | 0.0311 | 0.8548 |
|  | 509.02 | 60.37 | 295.79 | 0.059 | 0.0234 | 0.8240 |
|  | 763.53 | 77.62 | 542.82 | 0.046 | 0.0197 | 0.7996 |
|  | 1018.04 | 91.93 | 844.52 | 0.039 | 0.0173 | 0.7708 |
|  | 1272.55 | 103.82 | 1195.94 | 0.034 | 0.0155 | 0.7432 |
|  | 1527.06 | 114.20 | 1593.39 | 0.031 | 0.0141 | 0.7107 |
|  | 1781.57 | 123.31 | 2034.14 | 0.029 | 0.01296 | 0.6964 |
|  | 2036.07 | 131.35 | 2516.06 | 0.027 | 0.01202 | 0.6757 |
|  | 2290.58 | 138.41 | 3037.32 | 0.026 | 0.0112 | 0.65660 |
|  | 2545.09 | 144.84 | 3596.48 | 0.025 | 0.0105 | 0.63769 |
| **SMC1.5** | 254.51 | 37.34 | 78.42 | 0.095 | 0.0298 | 0.92210 |
|  | 509.02 | 57.36 | 210.46 | 0.062 | 0.0221 | 0.87689 |
|  | 763.53 | 73.44 | 384.22 | 0.048 | 0.0186 | 0.84898 |
|  | 1018.04 | 86.72 | 594.91 | 0.041 | 0.0162 | 0.81721 |
|  | 1272.55 | 98.02 | 839.02 | 0.036 | 0.0146 | 0.78969 |
|  | 1527.06 | 107.57 | 1113.90 | 0.033 | 0.0132 | 0.75428 |
|  | 1781.57 | 115.95 | 1417.53 | 0.031 | 0.0121 | 0.73864 |
|  | 2036.07 | 123.50 | 1748.33 | 0.029 | 0.0113 | 0.71722 |
|  | 2290.58 | 130.16 | 2104.99 | 0.028 | 0.0105 | 0.69777 |
|  | 2545.09 | 136.51 | 2486.45 | 0.026 | 0.0099 | 0.67974 |
| **SMC2** | 254.51 | 36.27 | 61.76 | 0.098 | 0.0288 | 0.96981 |
|  | 509.02 | 55.18 | 165.12 | 0.064 | 0.0212 | 0.91478 |
|  | 763.53 | 70.38 | 299.87 | 0.051 | 0.0177 | 0.88373 |
|  | 1018.04 | 83.21 | 461.94 | 0.043 | 0.0155 | 0.85313 |
|  | 1272.55 | 94.30 | 648.60 | 0.038 | 0.01396 | 0.82783 |
|  | 1527.06 | 103.86 | 857.75 | 0.034 | 0.01273 | 0.79453 |
|  | 1781.57 | 112.40 | 1087.81 | 0.032 | 0.01174 | 0.78209 |
|  | 2036.07 | 119.96 | 1337.58 | 0.030 | 0.01092 | 0.76175 |
|  | 2290.58 | 126.89 | 1606.00 | 0.028 | 0.01022 | 0.74444 |
|  | 2545.09 | 133.22 | 1892.27 | 0.027 | 0.0096 | 0.72659 |
