# Supplementary material for: Numerical study on flow and heat transfer characteristics of rectangular mini-channel of interpolated double S turbulators
Source: PLoS One. 2024 Feb 15;19(2):e0297678. doi: 10.1371/journal.pone.0297678 (PMC10868837; doi:10.1371/journal.pone.0297678)
Supplement: S2 Table — (DOCX) [file pone.0297678.s002.docx]

S2 Table. Local data supplement

| Re | x^*^ | Nu_x_ | | | |
| --- | --- | --- | --- | --- | --- |
|  |  | SMC | SMC1 | SMC1.5 | SMC2 |
| 254.51 | 1.55E-04 | 113.61 | 123.43 | 128.78 | 128.22 |
|  | 3.07E-04 | 54.24 | 57.89 | 82.43 | 78.66 |
|  | 4.61E-04 | 37.75 | 44.53 | 29.84 | 67.22 |
|  | 6.15E-04 | 26.06 | 58.75 | 42.77 | 18.37 |
|  | 7.69E-04 | 25.11 | 43.19 | 60.72 | 42.04 |
|  | 9.23E-04 | 22.10 | 68.59 | 36.36 | 50.00 |
|  | 0.00108 | 17.61 | 43.20 | 44.93 | 55.05 |
|  | 0.00123 | 18.48 | 65.76 | 60.79 | 24.55 |
|  | 0.00139 | 17.98 | 41.45 | 43.25 | 46.43 |
|  | 0.00154 | 14.66 | 66.08 | 43.59 | 42.34 |
|  | 0.0017 | 16.20 | 38.48 | 59.08 | 55.22 |
|  | 0.00185 | 14.92 | 77.09 | 62.85 | 56.24 |
|  | 0.00201 | 4.29 | 71.00 | 62.67 | 64.08 |
| 1018.04 | 7.71E-05 | 171.78 | 178.11 | 183.96 | 184.02 |
|  | 1.53E-04 | 82.31 | 92.44 | 122.77 | 116.75 |
|  | 2.29E-04 | 59.26 | 61.74 | 52.52 | 99.43 |
|  | 3.05E-04 | 42.58 | 91.52 | 60.80 | 38.80 |
|  | 3.81E-04 | 40.54 | 60.64 | 89.97 | 63.49 |
|  | 4.60E-04 | 35.86 | 109.99 | 63.45 | 79.02 |
|  | 5.36E-04 | 28.43 | 63.66 | 57.96 | 84.85 |
|  | 6.14E-04 | 28.66 | 105.96 | 95.58 | 37.77 |
|  | 6.91E-04 | 26.26 | 61.22 | 75.87 | 67.10 |
|  | 7.67E-04 | 22.47 | 108.33 | 59.07 | 72.64 |
|  | 8.45E-04 | 22.62 | 55.46 | 96.35 | 87.18 |
|  | 9.22E-04 | 22.76 | 124.30 | 103.60 | 86.45 |
|  | 9.98E-04 | 5.38 | 94.29 | 75.95 | 73.47 |
| 1527.06 | 5.12E-05 | 217.23 | 212.59 | 222.93 | 223.00 |
|  | 1.02E-04 | 105.63 | 120.99 | 149.67 | 144.36 |
|  | 1.53E-04 | 74.63 | 73.18 | 71.88 | 124.51 |
|  | 2.04E-04 | 55.27 | 112.57 | 72.85 | 53.20 |
|  | 2.54E-04 | 52.10 | 72.04 | 109.28 | 77.99 |
|  | 3.05E-04 | 46.95 | 139.40 | 84.87 | 100.07 |
|  | 3.55E-04 | 37.86 | 76.90 | 66.96 | 111.70 |
|  | 4.08E-04 | 38.13 | 135.31 | 116.78 | 46.92 |
|  | 4.59E-04 | 34.55 | 76.02 | 101.32 | 79.13 |
|  | 5.10E-04 | 28.97 | 138.83 | 70.68 | 92.48 |
|  | 5.62E-04 | 31.07 | 66.62 | 121.33 | 112.75 |
|  | 6.13E-04 | 29.30 | 159.54 | 136.99 | 108.87 |
|  | 6.64E-04 | 7.84 | 111.34 | 81.78 | 83.16 |
| 2036.07 | 3.83E-05 | 258.17 | 241.59 | 248.48 | 248.55 |
|  | 7.63E-05 | 123.05 | 139.05 | 174.39 | 167.39 |
|  | 1.14E-04 | 88.85 | 82.36 | 85.22 | 144.89 |
|  | 1.53E-04 | 63.09 | 128.92 | 81.41 | 62.65 |
|  | 1.91E-04 | 63.65 | 79.93 | 123.86 | 92.55 |
|  | 2.29E-04 | 56.78 | 162.57 | 101.15 | 113.66 |
|  | 2.67E-04 | 44.75 | 87.74 | 72.42 | 133.24 |
|  | 3.04E-04 | 45.04 | 155.54 | 136.84 | 56.05 |
|  | 3.42E-04 | 45.32 | 88.20 | 121.28 | 87.50 |
|  | 3.80E-04 | 35.45 | 163.72 | 75.88 | 109.60 |
|  | 4.21E-04 | 35.63 | 75.20 | 143.50 | 132.72 |
|  | 4.59E-04 | 35.81 | 186.04 | 160.20 | 127.08 |
|  | 4.97E-04 | 10.26 | 125.89 | 88.45 | 90.62 |
